# Potential of E3 Ubiquitin Ligases in Cancer Immunity: Opportunities and Challenges

**DOI:** 10.3390/cells10123309

**Published:** 2021-11-25

**Authors:** Peng Ye, Xiaoxia Chi, Jong-Ho Cha, Shahang Luo, Guanghui Yang, Xiuwen Yan, Wen-Hao Yang

**Affiliations:** 1 Key Laboratory of Cell Homeostasis and Cancer Research of Guangdong Higher Education Institutes and Affiliated Cancer Hospital & Institute, Guangzhou Medical University, Guangzhou 910095, China; 2019217972@stu.gzhmu.edu.cn (P.Y.); xiaoxia@stu.gzhmu.edu.cn (X.C.); ShahangLuo@stu.gzhmu.edu.cn (S.L.); GuanghuiYang@stu.gzhmu.edu.cn (G.Y.); 2 Department of Biomedical Science and Engineering, Graduate School, Inha University, Incheon 22212, Korea; chajongho@inha.ac.kr; 3 Department of Biomedical Sciences, College of Medicine, Inha University, Incheon 22212, Korea; 4 Graduate Institute of Biomedical Sciences, China Medical University, Taichung 406040, Taiwan

**Keywords:** E3 ubiquitin ligase, cancer immunotherapy, immune checkpoints, immune signaling pathway

## Abstract

Cancer immunotherapies, including immune checkpoint inhibitors and immune pathway–targeted therapies, are promising clinical strategies for treating cancer. However, drug resistance and adverse reactions remain the main challenges for immunotherapy management. The future direction of immunotherapy is mainly to reduce side effects and improve the treatment response rate by finding new targets and new methods of combination therapy. Ubiquitination plays a crucial role in regulating the degradation of immune checkpoints and the activation of immune-related pathways. Some drugs that target E3 ubiquitin ligases have exhibited beneficial effects in preclinical and clinical antitumor treatments. In this review, we discuss mechanisms through which E3 ligases regulate tumor immune checkpoints and immune-related pathways as well as the opportunities and challenges for integrating E3 ligases targeting drugs into cancer immunotherapy.

## 1. Introduction

**Figure 1:**
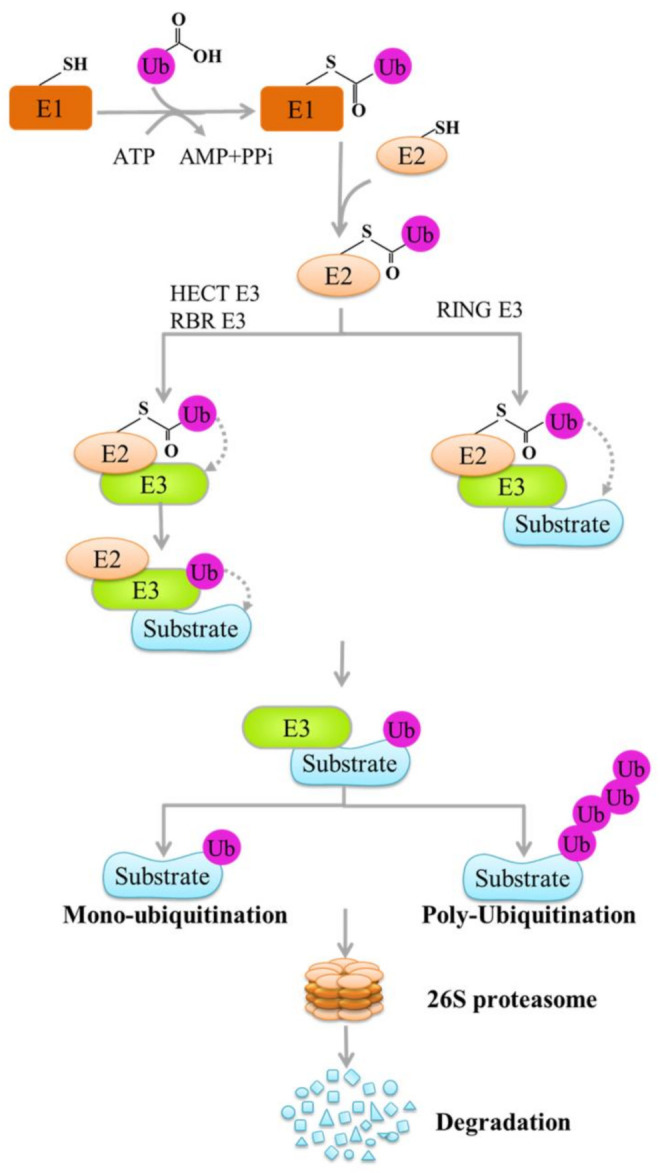
Schematic of the ubiquitination mechanism. Protein ubiquitination is catalyzed by the signaling cascades of ubiquitin-activating enzyme (E1), ubiquitin-conjugating enzyme (E2), and ubiquitin ligase (E3). In the case of ATP energy supply, E1 activates ubiquitin and transmits the activated ubiquitin to E2. Subsequently, the activated ubiquitin molecules are recruited to E3 ligases, E3 ligases connect ubiquitin to the target protein, and the target protein is ubiquitinated to be specifically recognized and degraded by the 26S proteasome. Some of the graphics in this figure was created using ScienceSlides software 2016 edition (VisiScience Inc., Chapel Hill, NC, USA).

Ubiquitination is a classical type of protein posttranslational modification [1,2]. In this process, ubiquitin (a highly conserved 76-amino-acid polypeptide) covalently binds to the substrate and mediates its transfer to the 26S proteasome complex for degradation [3,4]. Ubiquitination is catalyzed by the signaling cascades of ubiquitin-activating enzyme (E1), ubiquitin-conjugating enzyme (E2), and ubiquitin ligase (E3) [5] and leads to the covalent binding of ubiquitin to the target protein [6]. Ubiquitin is activated in an ATP-dependent manner and binds to the cysteine residue on E1 through a thioester bond. Subsequently, the activated ubiquitin molecule is transferred to the cysteine active site of E2 and then recruited into E3 ligases. Finally, E3 ligases bind the E2-Ub complex to the target protein and promote the binding of ubiquitin to the lysine residues of the substrate (Figure 1) [4,6,7]. Ubiquitin molecules can not only undergo monoubiquitination with seven lysine residues (Lys6, Lys11, Lys27, Lys29, Lys33, Lys48, and Lys63) but also form polyubiquitin chains based on the original target substrate [8,9]. Monoubiquitination is the addition of a ubiquitin molecule to a lysine residue, and polyubiquitination is the formation of a ubiquitin chain on a single residue on the substrate [10]. In the polyubiquitination chain, Lys48- and Lys11-linked polyubiquitination is related to proteasome degradation, and Lys63-linked polyubiquitination can participate in cell signal assembly and transduction [11]. In addition to serving as a signal for specific subcellular localization and mediating protein–protein interactions, monoubiquitination is also related to the degradation of a few proteins [10]. Monoubiquitination targets proteins with less than 150 amino acids and low structural disorder, while polyubiquitination recognizes proteins of any size with a highly disordered structure [10]. Ubiquitination is closely related to various biological processes, including internalization and lysosomal targeting, protein interaction regulation, changes in subcellular distribution, transcriptional regulation, growth response, innate immune response, DNA repair and replication, and transmembrane signal transduction, particularly in the regulation of the nuclear factor kappa B (NF-κB) pathway [4,12].

E3 ubiquitin ligases, critical enzymes in the ubiquitination reaction, mediate the recognition of the substrate in the ubiquitin–proteasome system and determine the specificity of the ubiquitination reaction [13]. On the basis of their structural characteristics and action mechanism, they are classified into three families: the really interesting new gene (RING) domain, the homologous to the E6-associated protein carboxyl terminus (HECT) domain, and the RING-between-RING (RBR) types of E3 ligases [1,3,14]. After the E3 ligases bind to the E2-Ub complex and the substrate, the RING-type E3 ligases catalyze the direct transfer of ubiquitin from E2 to the lysine residue of the substrate [1]. However, HECT and RBR E3 ligases catalyze the transfer of ubiquitin from E2 to cysteine, the active site of E3 ligases. Subsequently, ubiquitin is transferred to substrate proteins (Figure 1) [15]. E3 ligases play a vital role in the occurrence and progression of human cancer [2,16].

Cancer immunotherapy is becoming increasingly crucial in the field of tumor therapy and has shown promising results in the treatment of various advanced malignant tumors [13,17]. With the emergence of cancer immunotherapy, the progression-free survival and overall survival of patients with cancer have improved; however, drug resistance and adverse reactions remain the main challenges of immunotherapy management [18]. The future direction of immunotherapy is to identify new targets and develop combination therapy and new methods for reducing side effects [19]. An increasing number of E3 ligases are being identified as the crucial regulators of tumor immune responses, including the membrane-associated RING-CH (MARCH) family of E3 ligases [5] and F-box only protein 38 (FBXO38), which mediate the proteasomal degradation of programmed cell death protein 1 (PD-1) [20,21]. Targeting E3 ligases can enhance antitumor immunity in cancer immunotherapy [13]. Therefore, targeting E3 ligases may be an effective strategy for clinical cancer immunotherapy. However, the specific mechanism of E3 ligases in cancer cells under the background of drug resistance in cancer immunotherapy remains unclear. Many researchers have attempted to determine the role of E3 ligases in tumor immunity and its potential mechanism.

In this review, we focus on the role of E3 ligases in immune response and their potential in cancer immunity, including immune checkpoints and immune-related signaling pathways. In addition, we summarize the research and development of targeted selected E3 ligase drugs and their potential mechanisms in tumor immunity.

## 2. E3 Ligases and Tumor Immune Checkpoints

Immune checkpoint blockade (ICB) is an effective strategy for enhancing antitumor immune activity and clinical efficacy [22]. Inhibitors blocking the interaction of immune checkpoints have resulted in a breakthrough in cancer immunotherapy, and these inhibitors have substantial potential to enhance antitumor immunity [23]. Although immune checkpoint inhibitors have resulted in a durable clinical response in various cancers, only some patients with cancer can benefit from treatment [23,24,25]. In addition, immune checkpoint inhibitors cause severe immune-related adverse events [22,24,26]. Simultaneously, ICB resistance is an inevitable obstacle to durable antitumor activity [27]. Therefore, new small molecules, peptides, or compounds that can effectively target immune checkpoints are urgently required to maximize the efficacy of ICB in cancer treatment. E3 ligases play a vital role in regulating immune checkpoints [20,21,28,29,30]. Thus, an in-depth investigation of the effect of E3 ligases on tumor immune checkpoints may help discover alternative methods that can target immune checkpoints to enhance antitumor immunity and catalyze the development of new immune checkpoint regulators.

### 2.1. PD-1/Programmed Cell Death Protein Ligand 1 (PD-L1)

The PD-1/programmed cell death protein ligand 1 (PD-L1) signaling pathway plays a crucial role in tumors evading immune surveillance [31]. Tumors negatively regulate immune response by expressing PD-L1, which interacts with PD-1 on T-cells. PD-1 binds to PD-L1 to activate downstream signaling pathways and inhibit T-cell activation, thus inhibiting the specific killing of tumor cells by T-cells and resulting in tumor immune escape [32]. PD-1/PD-L1 signaling is essential for cancer immune evasion and has become the main target of anticancer immunotherapy. PD-1 is a cell surface receptor that is highly expressed on activated T-cells, B-cells, monocytes, dendritic cells (DCs), regulatory T-cells (Tregs), and natural killer (NK) T-cells (NKTs) [33,34]. PD-L1 is highly expressed on certain types of tumor cells and antigen-presenting cells (APCs) [26,34,35].

Lys48-linked polyubiquitination modification, the first protein modification after the translation of PD-1, can regulate the PD-1 protein level on the surface of activated T-cells and antitumor immunity [30]. FBXO38, a multiprotein complex belonging to the SKP1-CUL1-F-box protein (SCF) family of ligases, is a specific E3 ligase of PD-1 protein [21]. It mediates the Lys48-linked polyubiquitination of the Lys233 residue in the cytoplasmic domain of PD-1 protein and subsequent proteasome degradation [20,21], thus downregulating the surface PD-1 expression and blocking tumor immune escape mediated by PD-1/PD-L1. These findings indicate that FBXO38 regulates antitumor immunity by degrading PD-1 [20] and propose a novel possibility of the PD-1 pathway blockade, making FBXO38 serve as a potential target for the development of antitumor immune agents. Lyle et al. demonstrated that Casitas B lymphoma (c-Cbl) is a novel E3 ligase of PD-1 [28]. They observed that the C terminus of c-Cbl in immune cells (such as macrophages) binds to the cytoplasmic tail of PD-1 and ubiquitinates PD-1 to target it for ubiquitination-proteasome degradation. This process downregulates PD-1 expression and enhances tumor cell phagocytosis, eventually restricting tumor growth [28].

Both mono- and polyubiquitination regulate the expression, membrane location, and function of PD-L1 [30]. The immunosuppressive activity of PD-L1 is strictly regulated by ubiquitination and N-glycosylation [36]. After glycosylation, PD-L1 is transferred to the cell surface, whereas nonglycosylated PD-L1 is phosphorylated through glycogen synthase kinase 3β (GSK3β), triggering it to interact with the Cullin-RING-type E3 ligase ligand protein β-TrCP and thus resulting in the promotion of PD-L1 polyubiquitination and subsequent proteasome-dependent degradation [30,36]. Cullin3^SPOP^, a member of the Cullin-RING E3 ligase family, binds to the C-terminal tail of PD-L1 protein to promote the degradation of PD-L1 protein [37]. In addition, the CBL family regulates PD-L1. For example, the E3 ligases Cbl-b and c-Cbl inhibit PD-L1 by inactivating PI3K/Akt, Janus kinase-signal transducer and activator of transcription (JAK-STAT), and MAPK-Erk signals, resulting in the downregulation of PD-L1 protein expression [29]. In addition, in Cbl-b–deficient mouse models, T and NK cells exhibited functional resistance to PD-1/PD-L1 regulation [38,39], implying that Cbl-b inhibition may act as an antitumor immune enhancer of the PD-1/PD-L1 axis.

**Figure 2:**
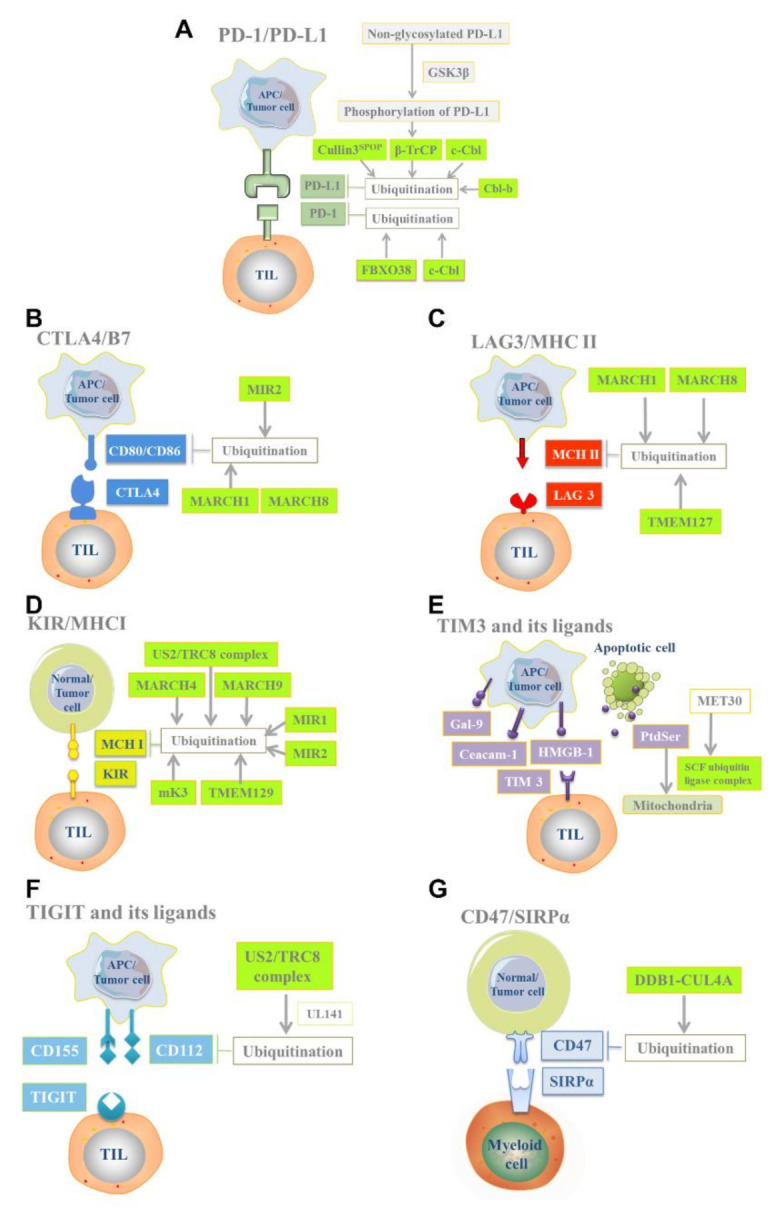
Schematic of E3 ligases regulating immune checkpoint ubiquitination. (**A**) Glycogen synthase kinase 3β (GSK3β) promotes the phosphorylation of nonglycosylated PD-L1 on the surface of antigen-presenting cells and tumor cells. Next, β-TrCP E3 ligase regulates the ubiquitination and degradation of PD-L1 protein. In addition, Cullin3SPOP, Cbl-b, and c-Cbl can bind to and degrade PD-L1 protein. FBXO38 and c-Cbl can degrade the PD-1 protein of tumor-infiltrating lymphocytes. (**B**) The E3 ligases MIR2, MARCH1, and MARCH8 mediate the degradation of CD86 protein. (**C**) The E3 ligases MARCH1, MARCH8, and TMEM127 mediate the degradation of MCH-II molecules. (**D**) The E3 ligases MIR 1, MIR 2, MARCH4, MARCH9, mK3, and US2/TRC8 complexes mediate the degradation of MCH-I molecules. (**E**) The SCF ubiquitin ligase complex mediates the intracellular transport of PtdSer (one of the ligands of TIM-3). (**F**) The US2/TRC8 complex mediates the degradation of CD112 (one of the ligands of TIGIT). (**G**) The DDB1-CUL4A E3 ligase mediates the degradation of CD47 protein. The Figure includes some elements from Servier Medical Art, licensed under a Creative Commons Attribution 3.0 Unported License.

Taken together, E3 ligases are crucial for PD-1/PD-L1 degradation (Figure 2A) and targeting them may sensitize patients with cancer to tumor ICB therapy (ICT) and enhance the efficacy of immunotherapy. Future studies investigating the interaction of E3 ligases with the tumor intrinsic PD-1/PD-L1 signaling pathway may provide new insights into ICT and facilitate the development of more effective combined immunotherapy to improve the efficacy of immunotherapy.

### 2.2. CTLA4/B7

Cytotoxic T lymphocyte–associated antigen 4 (CTLA-4, or CD152), a key negative immune checkpoint protein, is an inhibitory receptor of the CD28 immunoglobulin subfamily. CTLA-4 binds to its ligands CD80 (B7-1) and CD86 (B7-2) and inhibits T-cell activation [40,41], negatively regulates T-cell functions, and mediates tumor immunosuppression [42]. It is mainly expressed on the surface of activated T-cells, especially Tregs [41,43]. Its ligands CD80 and CD86 are usually present on the surface of APCs, such as DCs and B-cells [41,44]. Given their critical role in immune regulation, CTLA-4/B7 immune checkpoints can be targeted in cancer immunotherapy.

E3 ligases involved in CTLA-4 protein degradation remain unknown. Preliminary evidence has indicated a potential relationship between CTLA-4 protein abundance and E3 ligases [45]. A study reported that CTLA-4/B7 interaction promoted Cbl-b expression, indicating that CTLA-4 controlled T-cell activation and proliferation, at least in part, by regulating Cbl-b expression [46]. Ring finger protein 128 (RNF128, also known as GRAIL), a transmembrane E3 ligase, is highly expressed on Tregs [47]. It is associated with T-cell tolerance and is upregulated in CD4^+^ T-cells in the acute phase of infection, which may be related to a significant increase in inhibitory receptors (such as PD-1 and CTLA-4) expressed by CD4^+^ T-cells [48]. Moreover, in a T-cell malignancy, the polyubiquitinated proteins significantly increase and activate GATA3 (a T-cell transcription factor). Impaired proteasome function activates GATA3 in T-cells and upregulates CTLA-4 expression, thus inhibiting the T-cell response [49]. This study indicated that changes in proteasome function caused by an increase in the levels of polyubiquitinated proteins in T-cells may indirectly affect CTLA-4 expression, thus mediating tumor evasion from immune surveillance.

The MARCH protein, a subfamily of RING E3 ligases [50], is a critical regulator of immune responses [5]. Kaposi’s sarcoma herpesvirus modulator of immune recognition 2 (MIR2), a new type of membrane-bound E3 ligases, can degrade CD86 by inducing B-cell endocytosis, resulting in immune escape [51]. Furthermore, MIR2 ubiquitinates CD86 and induces CD86 to be endocytosed for degradation in immune cells [52]. A recent study reported that MARCH1 upregulation was negatively correlated with CD86 expression. MARCH1 E3 ligase mediates the ubiquitination and degradation of CD86 in DCs [53], which is crucial for regulating the antigen presentation of DCs. In addition, MARCH8 mediates the polyubiquitination of CD86 at its C terminus, leading to its rapid endocytosis and lysosomal-dependent degradation [5]. These findings indicate that CTLA-4 and its ligands are regulated by E3 ligases in some cases (Figure 2B), thus possibly providing a new therapeutic target for tumor immunosuppression by the CTLA4/B7 axis.

### 2.3. Lymphocyte Activation Gene-3 (LAG-3)/Major Histocompatibility Complex-II (MHC-II)

Lymphocyte activation gene-3 (LAG-3, or CD223) is a member of the immunoglobulin superfamily and is believed to regulate the immune response of T-cells [54]. LAG-3 is an inhibitory molecule mainly expressed in various immune cells, such as CD4^+^ and CD8^+^ T-cells, NKT cells, NK cells, plasmacytoid dendritic cells (pDCs), and Tregs [30,55,56]. Major histocompatibility complex (MHC)-II molecules are the classic ligands of LAG-3. MHC-II is constitutively expressed by immune cells and is mainly expressed on APCs such as B-cells, DCs, and macrophages [30,57]. MHC-II binds to a conservative extension loop in the D1 domain of LAG-3 protein and transmits inhibitory signals through its cytoplasmic domain, thus promoting tumor immune escape [58,59]. Therefore, blocking the LAG-3/MHC-II interaction may be an effective strategy to inhibit tumor immune escape.

Grosso et al. reported that LAG-3 was upregulated on the surface of tumor-infiltrating CD8^+^ T-cells. Moreover, the inhibition of LAG-3 restored the immune activity of CD8^+^ T-cells, suggesting that LAG-3 blockade may be a promising strategy for cancer treatment to promote tumor immunity [60]. However, the understanding of LAG-3 protein regulation currently remains limited, especially its posttranslational ubiquitination modification. Therefore, future studies should explore E3 ligases that mediate LAG-3 ubiquitination modification and elucidate the detailed mechanism through which E3 ligases regulate LAG-3 immune checkpoint protein.

The surface expression and half-life of MHC-II are controlled by ubiquitination [61,62,63]. The MARCH E3 ligase family, including MARCH1 and MARCH8, is considered to inhibit MHC-II expression [57,63,64]. Both MARCHs modulate the peptide-MHC-II complex by ubiquitinating the cytoplasmic tail of the MHC-II β chain [65]. MARCH8 ubiquitinates the lysine residue at position 225 in the IA β chain of MHC-II and downregulates the expression of MHC-II molecules on the cell surface, thus inhibiting the activation and function of T-cells [50,64,66,67,68,69]. In addition, MARCH1 mediates the ubiquitination of MHC-II molecules on the surface of DCs on the lysine 225 of its cytoplasmic domain [62,63], thus reducing their stability on the cell surface [70]. This ubiquitination induces MHC-II endocytosis and degradation [62,71,72], inhibiting antigen presentation and prompting tumor cells to escape immune destruction. Another study demonstrated that the immune process could be inhibited by degrading MHC-II through ubiquitination. The researchers observed that E3 ligases, including the tumor suppressor TMEM127 and WW domain-containing E3 ubiquitin protein ligase 2 (WWP2), regulate the ubiquitination and degradation of MHC-II. In their research, *Salmonella enterica* effector (SteD) mediates the interaction between the TMEM127/WWP2 complex and MHC-II, leading to the ubiquitination and degradation of MHC-II on the DCs surface and suppressing MHCII-dependent CD4+ T-cell proliferation and activation, which suppress the immune response [73]. These findings indicate that WWP2, MARCH1, and MARCH8 might be new targets for blocking the LAG-3/MHC-II immune checkpoint pathway (Figure 2C). Future studies determining the E3 ligases that mediate LAG-3/MHC-II expression and function regulation can help elucidate the role of E3 ligases in tumor immune response and develop related targeted drugs to strengthen immune surveillance.

### 2.4. Killer Cell Immunoglobulin-like Receptor (KIR)/MHC-I

Killer cell immunoglobulin-like receptors (KIRs, or CD158) are a family of transmembrane glycoproteins expressed on NK cells and T-cell subsets [74]. KIRs can be divided into three subgroups: inhibitory receptors, activating receptors, and a unique activating receptor called KIR2DL4, which plays a vital role in the regulation of NK cell function [75]. The main ligands of KIRs are MHC-I (also known as human leukocyte antigen class I, HLA-I) molecules, which are widely expressed in normal cells. The interaction between MHC-I and inhibitory KIRs can inhibit NK cell activation and induce autoimmune tolerance [76,77]. Abnormal cells with downregulated or complete loss of MHC-I expression are vulnerable to attack by NK cells [78]. A high expression of inhibitory KIRs was associated with a poor prognosis in many patients with malignant tumors, and the absence or decreased expression of inhibitory KIRs and MHC-I molecules was associated with more favorable therapeutic effects [77]. Hence, we speculate that E3 ligases might play a role in the targeted degradation of KIRs and MHC-I molecules.

The K1 gene products of KSHV, such as MIR1 and MIR2, are E3 ligases, and MHC-I molecules present on the cell surface are degraded by lysine-63-linked polyubiquitin chains. [79]. The US2/TRC8 complex and TMEM129 E3 ligases mediate the ubiquitination and degradation of MHC-I molecules [80]. Several MARCH proteins downregulate the cell surface expression of MHC-I molecules. For example, MHC-II ubiquitination mediated by MARCH1 reduces surface MHC-I expression and affects MHC-I antigen presentation [63]. MARCH4 and MARCH9 directly mediate the ubiquitination of MHC-I molecules, resulting in their endocytosis and lysosomal degradation (Figure 2D) [5]. Therefore, regulating the expression of MHC-I molecules in tumors by targeting E3 ligases may be a promising strategy for antitumor immunotherapy.

### 2.5. T-Cell Immunoglobulin and Mucin Domain-3 (TIM-3)

T-cell immunoglobulin and mucin domain-3 (TIM3), an inhibitory checkpoint protein, is a member of the TIM immunomodulatory protein family [81]. TIM3 contains an immunoglobulin domain (IgV), a membrane-proximal mucin-like domain containing an O-linked glycosylation site (a glycosylated mucin domain), a transmembrane region, and a C-terminal cytoplasmic tail with five conserved tyrosine signaling motifs [82]. TIM-3 is expressed in CD4^+^ T-cells, CD8^+^ T-cells, NK cells, APCs, and myeloid cell lineages [83]. Four ligands related to TIM3 bind to the IgV domain of TIM-3, including galectin 9 (Gal-9), high-mobility group protein B1 (HMGB1), carcinoembryonic antigen cell adhesion molecule 1 (CEACAM-1), and phosphatidylserine (PtdSer) [81,84,85]. TIM-3 blockade could increase the cell-mediated antitumor immune response and inhibit tumor growth [84].

The specific mechanism through which TIM-3 promotes or inhibits the cellular immune response depends on the ligand model involved and the cellular environment [86,87,88]. Although the exact E3 ligase that degrades TIM-3 and its ligands has not yet been discovered, a potential relationship between that E3 ligase and TIM-3 and its ligands might affect tumor immunity. It has been found that the transport of PtdSer, one of the TIM-3 ligands, is regulated by ubiquitination, which is mediated by the substrate recognition subunit of the SCF ubiquitin ligase complex encoded by the MET30 gene (Figure 2E) [89]. Therefore, targeting E3 ligases that regulate TIM-3 ligands may be an alternative strategy to enhance antitumor immunity.

### 2.6. T-Cell Ig and ITIM Domain (TIGIT) and Its Ligands CD155 and CD112

The T-cell Ig and ITIM domain (TIGIT) is an emerging immune checkpoint that can inhibit the antitumor immune response and, thus, has become a particularly attractive target for cancer immunotherapy [90]. TIGIT is an inhibitory immunoglobulin receptor that is related to tumor immune surveillance. The role of TIGIT in tumor immune surveillance is similar to that of the PD-1/PD-L1 axis in tumor immunosuppression [91]. TIGIT is expressed by activated T-cells, NK cells, and Tregs [92], and its ligands include CD112 (NECTIN2) and CD155 (Necl-5) [83], which are expressed by tumor cells and APCs within the tumor microenvironment (TME) [93,94]. TIGIT binds to its ligands and plays a crucial role in regulating antitumor immunity [95].

With the help of “holdase” UL141 encoded by human cytomegalovirus, the TRC8 (RNF139) E3 ligase can recognize and effectively degrade CD112 [80]. A recent study reported that in tumor cells, the ubiquitination modification of CD112 promoted its proteasome degradation and intracellular protein retention. Therefore, inhibiting CD112 ubiquitination can increase its cell surface expression, thus increasing the sensitivity of tumor cells to NK-mediated cytolysis and enhancing the killing effect of NK cells on tumor cells [96]. However, a study observed that TIGIT blockade or TIGIT knockdown alone exerted no substantial effect on tumor growth and metastasis [97]. In addition, TIGIT, TIM-3, LAG-3, and PD-1 inhibited the antitumor response through synergism to T-cell depletion [83]. Hence, a combined therapy aimed at the synergistic effects of these coinhibitory molecules may considerably improve the antitumor immunity effect of monotherapy. Because of the absence of recent research on TIGIT and its ligands, the exact relationship between them and E3 ligases remains unclear (Figure 2F). Future studies should identify E3 ligases that regulate several coinhibitory molecules to determine a promising new target for combined immunotherapy.

### 2.7. CD47/Signal Regulatory Protein α (SIRPα)

CD47/Signal regulatory protein α (SIRPα) is a crucial immune checkpoint pathway that is crucial for maintaining self-stability and eliminating tumor cells [98]. CD47 (also known as MER6 or OA3) is a transmembrane protein expressed on normal and cancer cells [99,100]. SIRPα is the main receptor of CD47 and an inhibitory immune checkpoint protein expressed on myeloid cells [100]. CD47 promotes cancer development by enabling tumor cells to escape phagocytosis [101]. The expression of CD47 on tumor cells inhibits myeloid cell-mediated elimination in a manner similar to that of PD-1/PD-L1 immune cell checkpoints that inhibit T-cell activity in tumors [99,102]. When CD47 binds to SIRPα, it transmits the “do not eat me” signal to macrophages, negatively regulates phagocytosis, and weakens the presentation of tumor antigens to T-cells, thus reducing the antitumor effect mediated by macrophages and cytotoxic CD8^+^ T-cells [103] and helping tumor cells escape immune surveillance and phagocytosis [104,105]. Currently, disrupting the CD47/SIRPα interaction has become a new antitumor immunotherapy strategy that induces the phagocytosis and elimination of tumor cells [106].

Cullin-RING ligase 4 (CRL4) is a member of the E3 ligase family that is composed of RING finger protein RBX1, CUL4 scaffold protein, and DDB1-CUL4-related substrate receptors [107]. CD47 is ubiquitinated by DDB1-CUL4A and then transported to the proteasome for degradation (Figure 2G) [30], thereby blocking the CD47/SIRPα signaling pathway and enhancing antitumor immunity. However, the specific mechanism of E3 ligases in the CD47/SIRPα signaling pathway remains unclear. This finding suggests that antitumor immunity can be enhanced by blocking the CD47/SIRPα signaling pathway by targeting E3 ligases. However, because of the widespread expression of CD47 in normal tissues, CD47 degradation therapy may cause off-target toxicity. Therefore, future studies examining CD47 ubiquitination to develop the most effective cancer treatment should consider the effect of related side effects on the prognosis of patients with cancer.

## 3. E3 Ligases and Immunomodulatory Pathways

Inflammation is a critical immune response that protects the host’s body from pathogens to maintain tissue homeostasis. However, inflammation can affect the development and treatment of tumors, thus promoting or inhibiting tumors. As a crucial risk factor for malignant tumors, chronic inflammation promotes the formation of the TME by releasing inflammatory mediators and activating immune pathways that ultimately lead to angiogenesis and antitumor immunity [1]. Some major inflammatory pathways, such as NF-κB and JAK-STAT signaling pathways, are involved in inflammation-induced carcinogenesis [108]. Moreover, the activation of the STING pathway can lead to antitumor T-cell responses. However, in some conditions, STING activation might facilitate inflammation-induced carcinogenesis [109]. E3 ligases play a vital role in regulating these immune-related pathways [1,110,111] by acting like a pathway switch, controlling the activation or inhibition of these pathways.

### 3.1. NF-κB Signaling Pathway

The NF-κB transcription factor family is a key participant not only in the innate and adaptive immune response but also in many steps of cancer occurrence and development [112,113]. NF-κB can mediate chronic inflammation by regulating many immune cells in the TME, thus regulating tumor activity. For example, NF-κB is involved in cancer cell proliferation and survival, cell apoptosis inhibition, epithelial–mesenchymal transition, invasive behavior, angiogenesis, metastasis, cancer stem cell formation, cell metabolism, and treatment resistance [114,115]. Tumor-associated macrophages (TAMs) are usually the most abundant immune cells in the TME [116]. Polarized TAMs are mainly of two types: M1 and M2. The M1 type is believed to enhance antitumor immunity and is induced and activated by interferon-γ (IFN-γ) and lipopolysaccharides (LPS). By contrast, the M2 type is believed to promote tumor growth and is induced by IL-4, IL-10, and IL-13. Blocking the activation of NF-κB in TAMs can transform the tumor-promoting M2 phenotype to the M1 cytotoxic phenotype, thus inhibiting tumor growth [117,118]. In addition, classic NF-κB activation in T-cells can increase the number of CD8^+^ T-cells required for eliminating tumors [119]. However, the activation of NF-κB promotes the development of CD4^+^CD25^+^ Treg cells and their immune suppression function. High infiltration of this group of cells in breast, cervical, and kidney cancer is often a poor prognostic marker [120]. Therefore, NF-κB pathway activation can affect different immunosuppressive cells and immune killer cells in the TME, thus promoting or inhibiting tumor growth.

**Figure 3:**
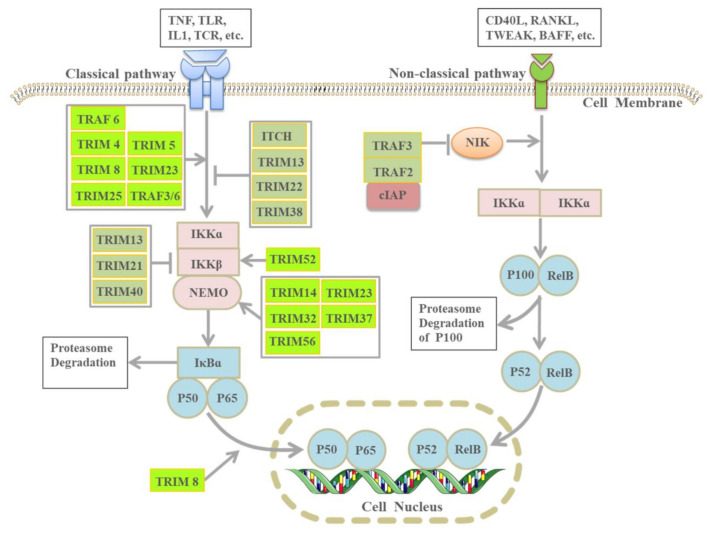
E3 ligases regulate NF-κB pathway activation. The classic NF-κB activation pathway is activated by TNF, TLR, IL-1, and TCR, and the noncanonical NF-κB activation pathway is activated by CD40L, RANKL, TWEAK, BAFF, LTβ, and LIGHT. The E3 ligase in the green module can promote NF-κB pathway activation, whereas the E3 ligase in the gray module inhibits NF-κB pathway activation. This Figure includes some elements from Servier Medical Art, licensed under a Creative Commons Attribution 3.0 Unported License.

E3 ligases are involved in the three steps of NF-κB signal activation: the degradation of the NF-κB inhibitor IκB, the processing of NF-κB precursors, and the activation of the IκB kinase (IKK) complex through a degradation-independent mechanism [121]. The NF-κB transcription factor family includes five members, namely, RelA (p65), RelB, c-Rel, NF-κB1 (p105), and NF-κB2 (p100). p105 and p100 are usually processed into their shorter forms, p50 and p52, respectively, which form dimers or heterodimers in different combinations [112]. These dimers bind to the IκB protein family (inhibitors of NF-κB), thus inactivating their function. Classical and nonclassical pathways are involved in NF-κB activation. The classic NF-κB pathway can be activated by various receptors, including tumor necrosis factor (TNF) receptor 1, Toll-like receptors (TLRs), interleukin (IL)-1 receptors, and T-cell receptors (TCRs) [122]. The classical pathway activates NF-κB by releasing IκB molecules, whereas the nonclassical pathway is activated by p100 and p105 cleaved into p50 or p52. The classic pathway is also called the NF-κB essential modulator (NEMO)–dependent pathway, which is mediated by a kinase complex composed of the NEMO and two IκB kinases (IKKα and IKKβ). The NEMO/IKK complex mediates IκB ubiquitination and proteasome degradation. After IκB is degraded or downregulated, the inhibition of NF-κB is reduced, and free NF-κB is subsequently transferred to the nucleus to activate the expression of related genes. In addition, the NEMO itself is a substrate of ubiquitination, and these mechanisms are mediated by the ubiquitin-binding domain of the NEMO (Figure 3) [113].

The nonclassical NF-κB activation pathway involves NF-κB-induced kinases (NIKs) and IKKα-dependent transduction. Ligands used to activate noncanonical NF-κB signaling pathways include the CD40 ligand (CD40L), NF-κB receptor activator ligand (RANKL), TNF-like weak apoptosis inducer (TWEAK), B-cell activation factor (BAFF), and lymphotoxin beta (LTβ) [114]. After these ligands bind to the receptor, NIK activates IKKα and phosphorylates p100. This phosphorylation triggers the K-48-linked ubiquitination and proteasome-mediated partial degradation of p100 to generate p52 and form the RelB–p52 complex. Subsequently, RelB–p52 heterodimers translocate to the nucleus and activate their target genes (Figure 3) [123].

TNF receptor-related factors (TRAFs) are a family of signaling molecules that play a key role in the biology of innate and adaptive immune cells. Except for TRAF1, which lacks the loop domain, all TRAFs have E3 ligase activity. Members of the TRAF family can positively or negatively regulate classical and noncanonical NF-κB signaling [124]. TRAF6 activates IL-1 and is related to TLR-mediated NF-κB pathway activation, whereas TRAF2/5 is related to TNFR1-mediated NF-κB pathway activation [125]. In addition, TRAF2 and TRAF3 are the key negative regulators of the nonclassical NF-κB pathway [126]. Under normal physiological conditions, NIKs usually bind to TRAF3. The TRAF–cIAP E3 ligase complex, composed of TRAF2, TRAF3, and cellular inhibitors of apoptosis (cIAPs), can bind to NIKs and degrade them through the TRAF3 protein, which can prevent NIKs from accumulating and activating noncanonical NF-κB signals. When receiving pathway activation signals, such as CD40L, BAFF, and RANKL, TRAF2 and cIAP1/2 mediate the degradation of TRAF3 and then release NIKs, thus leading to noncanonical NF-κB activation [127]. The tripartite motif (TRIM) is a typical E3 ligase, a multiple-member family involved in the ubiquitin-dependent proteolysis of the NF-κB signaling pathway. TRIM4, 5, 8, 14, 23, 25, 32, 37, 52, and 56 stably increase NF-κB activity, whereas TRIM13, 21, 22, 38, and 40 inhibit NF-κB activity (Figure 3) [128]. Moreover, itchy E3 ubiquitin protein ligase (ITCH) inhibits the NF-κB signal induced by TNF-α [129]. The NF-κB pathway, closely related to tumor immunity, can be precisely regulated by targeting E3 ligases to maintain tumor progression.

### 3.2. JAK-STAT Signaling Pathway

The JAK-STAT signaling pathway is an intracellular pathway in which cytokines and other molecules transmit signals from the cell membrane to the nucleus [130]. The JAK-STAT signaling pathway consists of three components: tyrosine kinase–related receptors that receive signals, tyrosine kinase JAK (four members) that transmits signals, and transcription factor STAT (seven members) that exerts effects [131]. The JAK-STAT signaling pathway is a key pathway for tumorigenesis, development, and immune escape, and STAT3 and STAT5 have attracted considerable attention in cancer biology [132]. In tumor cells, the constitutive activation of STAT3 suppresses the antitumor immune response by inhibiting the expression of Th1 mediators and inducing the production of multiple immunosuppressive factors, leading to tumor immune evasion and tumor progression [130,133,134]. STAT3 has become a crucial target for cancer immunotherapy and inhibiting STAT3 can help improve the effects of various immunotherapies [132]. STAT5 is a crucial immunoregulatory factor divided into two subtypes, STAT5a and STAT5b, and it plays a vital role in the function and development of Tregs. The activation of STAT5 inhibits tumor immunity and promotes tumor cell proliferation, invasion, and survival [135]. Therefore, the targeted inhibition of the JAK-STAT signaling pathway may play a key role in tumor immunotherapy.

The JAK-STAT pathway consists of three types of negative regulators: phosphatases (SHPs, CD45, and PTP1B/TC-PTP), suppressors of cytokine signaling (SOCS) proteins, and protein inhibitors of activated STAT (PIAS). SOCS protein exhibits the activity of E3 ligase, leading to the proteasome degradation of signal molecules (including cytokine receptors and JAK kinases) [136]. SOCS1 gene silencing enhances the antigen presentation and antitumor immunity of DCs [137]. PIAS protein can bind to the E2 ligase Ubc9 and undergo SUMO modification through the RING finger domain to exhibit E3 ligase activity, leading to the degradation of the target protein STAT dimer and inhibiting the activation of JAK-STAT. PIAS3 is a specific inhibitor of STAT3 [138]. Therefore, enhancing or inhibiting the effects of these negative regulatory proteins can block the escape of tumor immune cells mediated by the JAK-STAT signal and improve the efficacy of tumor immunotherapy.

Although STAT3 and STAT5 are related to tumor immunosuppression, other members (such as STAT1) can mediate antitumor immunity, which is in contrast to the effects of STAT3 and STAT5a/b. The activation of STAT1 usually indicates a more favorable prognosis [131]. Moreover, the phosphorylation of Stat1 is related to the polarization of M1 macrophages, which has been shown to inhibit the development of cancer [139,140]. However, STAT1 can exert contrasting effects on tumor progression. Another study reported that STAT1 could promote tumor growth by mediating tumor immunosuppression [141]. Thus, STAT1 may exert tumor-suppressing or tumor-promoting effects to regulate tumor activity. PIAS1 is a specific inhibitor of STAT1 signal transduction [138]. The E3 ligase Smad ubiquitination regulator factor-1 (Smurf1) promotes the degradation of STAT1 in the proteasome, negatively regulates IFN-γ signal transduction, and interferes with the immune response process [142]. The NK lytic–associated molecule (NGLAM), a member of the RBR E3 ligase family, ubiquitinates STAT1 and positively regulates transcriptional activity mediated by STAT1, thus promoting the binding of STAT1 to DNA and participating in the immune response [143]. In addition, it has been reported that another E3 ligase, STAT-interacting LIM protein (SLIM), can mediate the proteasome degradation of STAT1 protein and enhance its dephosphorylation [144]. Another study showed that RNF2 promoted lys33-linked polyubiquitination of STAT1, resulting in the separation of STAT1/STAT2 from DNA and inhibiting IFN signaling and antiviral response [145]. However, a recent study showed that RNF220, a member of the RNF family, can mediate the Lys63-linked polyubiquitination of STAT1, promote the interaction between STAT1 and JAK1, and activate the interferon signaling pathway [146]. Moreover, recent studies have also shown that the linear ubiquitin chain assembly complex (LUBAC) can linearly ubiquitinate the Lys511 and Lys652 residues of STAT1, thereby inhibiting the antiviral signal of IFN-STAT1 [147].

Currently, the mechanism through which E3 ligases regulate the JAK-STAT signaling pathway and affect tumor immunity remains unclear. Elucidation of these mechanisms is a major challenge in the future. Once the interaction of these mediators is fully understood, these E3 ligases can be used in tumor immunotherapy by upregulating or downregulating these mediators.

### 3.3. Stimulator of Interferon Gene (STING) Signaling Pathway

Stimulator of interferon gene (STING) is an adaptor transmembrane protein located in the endoplasmic reticulum and is a crucial innate immunosensor for tumor detection. The activation of the STING pathway in APCs leads to the production of IFN-β and the induction of CD8^+^ T-cells, thus initiating and leading to an adaptive anticancer immune response [148]. Currently, various polyubiquitinations have been found in the STING pathway, including the polyubiquitination of K63, K48, K11, and K27 [1]. The E3 ligases Trim56 and Trim32 increase STING-mediated IFN-β expression by catalyzing the polyubiquitination of STING’s K63 link [149,150]. After herpes simplex virus type 1 (HSV-1) infection, the knockdown of Trim32 in THP-1 cells inhibited the expression of TNF and IL-1β, indicating that Trim32 positively regulates STING [150]. By contrast, the E3 ligase RNF5 catalyzes the K48 polyubiquitination of STING, causing it to be degraded by the proteasome [151]. Another study showed that RNF26 catalyzes the K11 polyubiquitin chain of STING, which can protect STING from RNF5-mediated degradation [152]. The role of ubiquitination in the activation or inactivation of STING may be quite complex, and further work is needed to clarify the role of different types of E3 ligases in the STING pathway. Trim29 was also identified as the E3 ligase of STING by two studies. One of the studies found that using Trim29 to degrade STING in airway epithelial cells and myeloid dendritic cells could reduce the production of type I interferon, which is conducive to the Epstein-Barr virus (EBV) establishing persistent infection in both cell types [153]. Another study found that the lack of Trim29 will activate STING and increase type I interferon and proinflammatory cytokines, and *Trim29 −/−* mice are more resistant to lethal HSV-1 infection than WT mice [154]. Another study found that Trim30α causes the K-48 ubiquitination of STING and promotes its degradation and negatively regulates STING-mediated DNA virus, triggering signal transduction. Animal models show that *Trim30α −/−* mice are more resistant to HSV-1 infection than WT mice [155]. In general, the STING pathway promotes or inhibits tumor progression by regulating the activity of the immune system. Moreover, the STING pathway is regulated by E3 ligases. Therefore, for tumor treatment, E3 ligases can be targeted to regulate immune activity mediated by the STING pathway.

## 4. Therapeutic Targeting of E3 Ligases in Cancer Immunotherapy

E3 ligases play a crucial role in tumor immunity. E3 ligases can be used as a tumor promoter or suppressor. Considering its role in activating or inhibiting tumor immunity, targeting E3 ligases provides new ideas for the research and development of antitumor drugs. Drug development for E3 ligases has been a challenging research hotspot in recent years. Currently, antitumor drugs targeting E3 ligases are mainly divided into four categories according to their mechanism of action: targeted inhibitors of E3 ligases, targeted agonists of E3 ligases, proteolysis targeting chimeras (PROTACs), and molecular glues.

### 4.1. E3 Ligase Inhibitors

An increasing number of E3 ligase inhibitors have been developed and used in clinical trials. Here, we describe inhibitors that target the three protein families of E3 ligases (Table 1).

#### 4.1.1. RING-Type E3 Ligase Inhibitors

The RING-type E3 ligase inhibitors developed in recent years are mainly small-molecule inhibitors targeting CRLs [173,203]. CRLs composed of Cullin family proteins, RING proteins, linker proteins, and substrate recognition subunits are the largest family of E3 ligases that can regulate the degradation of many proteins with different functions and structures. They have currently emerged as popular targets for the development of antitumor drugs targeting E3 ligases [183,203]. Many inhibitors of RING finger E3 ligases have demonstrated considerable therapeutic potential in preclinical models and clinical trials of cancer immunotherapy or combination therapy, among which the most studied inhibitors include the IAP family, MDM2, pVHL, Skp2, and β-TrCP [159,164,182,183,204].

The overexpression of the IAP family in human cancer is related to poor prognosis and chemoresistance in many cancers [157,159,205]; therefore, IAP proteins are promising targets for cancer treatment. IAP antagonists are developed by imitating Smac/Diablo small molecules, the natural IAP antagonist, and promoting the proteasome-dependent degradation of cIAP1, cIAP2, and X-linked IAP [206]. The process of targeting IAP proteins with small Smac mimetics revealed a method of inducing apoptosis in cancer cells [207].

IAP antagonists play a vital role in promoting antitumor immunity [160]. A study reported that IAP antagonists, such as LCL161, induce the TNF-dependent apoptosis of cancer cells in multiple myeloma and promote antitumor immunity, which effectively stimulates antitumor immunity by enhancing innate and adaptive immune responses [156]. LCL161 has shown high safety and effectiveness in phase 1 clinical trials of patients with advanced solid tumors (NCT01098838). In addition, APG-1387 could induce tumor cell death and enhance innate antitumor immunity in HBV-positive hepatocellular carcinoma with a high expression of cIAP2 and stimulate adaptive immunity in vitro by reducing Treg differentiation and PD-1 expression [158]. The SMAC mimic Debio 1143 enhanced tumor-specific adaptive immunity induced by ablation radiotherapy [208]. In addition, IAP antagonists could significantly improve the antitumor effects of cells or other immune agents in combination therapy, including chimeric antigen receptor (CAR) T-cells, cytokine-induced killer cells, NKT cell inducers α-GalCer, TNF-α, and PD-1 blockade [13]. At present, several studies on the synergistic effect of IAP antagonists and other anticancer agents are in phase ½ clinical trials (NCT02649673, NCT04643405). IAP antagonists have shown considerable potential in antitumor immunity [156,158]. IAP inhibitors may become an efficient tumor immunomodulator in the future.

Murine double minute 2 (MDM2), an E3 ligase containing the RING domain, is a pivotal negative regulator of the tumor suppressor p53 [172]. MDM2 is overexpressed in several types of human tumors, especially in sarcomas [209]. MDM2 promotes tumor growth and progression by mediating p53 ubiquitination degradation and p53-independent carcinogenesis [172]. One study reported that MDM2 negatively regulates T-cell activation in a p53-independent manner [210]. In addition, Guo et al. indicated that activating p53 in the TME could overcome immunosuppression and enhance antitumor immunity [169]. Therefore, blocking the MDM2–p53 interaction and rebuilding p53 function can be a new cancer treatment strategy used in clinical tumor immunotherapy.

MDM2 inhibitors work by inhibiting the E3 ligase activity of MDM2 and interfering with the MDM2-p53 interaction [162,211]. Many MDM2 inhibitors, with unique structure, high efficiency, and no peptides, have been successfully designed and developed [162], including Nutlin-3a [170], HLI98 [171], MEL23, and MEL24 [172]. KRT-232 (AMG 232), a small-molecule inhibitor of MDM2, reduces IL-6 expression, enhances T-cell-mediated cancer cell killing, and exerts a strong antitumor effect [163,165]. Another MDM2 inhibitor, APG-115, can enhance antitumor immunity in the TME by increasing M1 macrophage polarization and T-cell activation and the anti-PD-1-mediated antitumor effect on mouse models of cancer immunotherapy [166]. Currently, some inhibitors targeting MDM2 are being tested in human clinical trials as new anticancer drugs, including RG7112 (RO5045337) [162], SAR405838 (MI-77301) [167], and idasanutlin (RG7388) [168]. The results of existing studies on MDM2 inhibitors reveal the involvement of an antitumor immune signal. However, the dose-limiting toxicity and drug resistance caused by p53 mutations are two major challenges in clinical trials [162]. Future clinical trials of MDM2 inhibitors should focus on these two major problems and design more efficient and more specific MDM2 inhibitors.

The VHL tumor suppressor protein (pVHL) is the substrate recognition component of E3 ligases that targets the hydroxylated hypoxia-inducible factor (HIF) α-subunit for ubiquitination and proteasome degradation under normoxic conditions. The VHL E3 ligase complex is a crucial target for cancer immunotherapy [204,212,213]. Disrupting the VHL/HIF-α interaction may affect the HIF-independent tumor suppressor function of pVHL. The small-molecule inhibitors currently developed for p-VHL/HIF-1α include compound 15, compound 7, and VH298 [173,174]. However, the specific mechanism through which these small-molecule inhibitors affect cancer immunotherapy remains unclear. Although the mechanism through which these small-molecule inhibitors affect cancer immunotherapy remains unclear, the loss of HIF-1α adversely affects CD8^+^ T-cell infiltration, resulting in the loss of antitumor activity in cancer immunotherapy models and finally leading to accelerated tumor growth [214]. These findings indicate that inhibiting the degradation or loss of HIF-1α is beneficial for enhancing antitumor immunity. Therefore, VHL inhibitors may enhance antitumor immunity by regulating the function of immune cells in the TME.

S phase kinase-associated protein 2 (Skp2) is an oncogenic protein that targets the degradation of tumor suppressor proteins [183]. It plays a crucial role in cancer development and progression. It is overexpressed in various human cancers [215] and is associated with poor cancer prognosis [7,173]. The SCF^Skp2^ complex performs its carcinogenic function by promoting the ubiquitination of substrates such as p27, FOXO1, p21, and p57 and subsequent proteasome degradation [181]. Therefore, Skp2 may be a crucial target for various cancers in which Skp2 is abnormally activated or overexpressed [7].

Among the developed selective small molecular inhibitors of Skp2, mechanisms through which different inhibitors exert their antitumor effects by inhibiting the activity of the Skp2 E3 ligase are different [173,215]. For example, Compound A blocks the assembly of Skp2 in the SCF complex [175]; C1, C2, C16, and C20 inhibit p27 ubiquitination by targeting the binding interface between Skp2–Cks1 and p27 [176]; Compound 25 inhibits the formation of the Skp2–Skp1 complex [177]; DT204 reduces Skp2 binding to Cullin-1 and Commd1 (Cullin-1 binding protein) [178]; and betulinic acid binds to Skp2, reducing its stability and the accumulation of its substrate protein [179]. Dioscin may be a promising multitarget drug candidate for treating various tumors and exerts an immunomodulatory effect [216]. It may be a new type of Skp2 inhibitor for cancer treatment and may have lower toxicity and fewer side effects than chemically synthesized Skp2 inhibitors [180,181]. Therefore, Skp2 inhibitors may exert a certain effect on cancer immunotherapy regulation. However, the role of targeting Skp2 in immunoregulation has not been effectively explored. Future studies on the role of Skp2 in tumor immunity can help clarify the potential mechanism of tumor immune escape and the antitumor effect of Skp2 inhibitors.

The F-box protein β-transducin repeat-containing protein (β-TrCP), which refers to the substrate recognition subunit of RING finger E3 ligases, can regulate the ubiquitination and degradation of various vital proteins, including tumor suppressors and oncogenic drivers. The role of β-TrCP in promoting or inhibiting tumors depends on the properties of the substrate targeted by β-TrCP [183]. β-Trcp is highly expressed in breast cancer, colon cancer, hepatoblastoma, pancreatic cancer, and melanoma [217]. According to these studies, β-TrCP is considered to be an oncoprotein [7,183]. Therefore, the use of small-molecule inhibitors targeting β-TrCP is a promising strategy for developing anticancer drugs.

Erioflorin may act as a β-TrCP inhibitor, stabilizing the tumor suppressor Pdcd4 by inhibiting the β-TrCP1/Pdcd4 interaction and thus exhibiting antitumor potential [182]. In addition, the inhibition of β-TrCP can enhance the antiproliferative effect of antitumor drugs on breast cancer cells, thus inhibiting NF-κB activity [218]. Although the β-TrCP E3 ligase plays a key role in regulating immune response [219], the mechanism of β-TrCP inhibitors in tumor immunity remains unclear. Therefore, future studies should analyze the regulation of targeted β-TrCP in tumor immunity.

#### 4.1.2. HECT-Type E3 Ligase Inhibitors

HECT-type E3 ligases are involved in many types of human cancers [16]. Therefore, targeting the HECT E3 ligase may be a potential treatment strategy for human cancers. However, compared with RING-type E3 ligases, studies on HECT-type E3 ligase inhibitors are few [3]. The neural precursor cell–expressed developmentally downregulated gene 4 (NEDD4) family is one of the most characteristic HECT-type E3 ligases, which include NEDD4, ITCH, WWP2, SMURF1, and SMURF2. These ligases play a key role in promoting the occurrence and progression of human cancer [220]. In addition, the members of the NEDD4 family are related to the host immune response. For example, NEDD4 promotes the ubiquitination and degradation of Cbl-b protein to disrupt the transmission of the TCR signaling pathway [221]. ITCH and WWP2 regulate T-cell differentiation by regulating the TCR signal [222]. ITCH could suppress immunity by inhibiting the NF-κB pathway induced by TNF-α [129]. Another study reported that WWP2 negatively regulated TLR3-mediated innate immunity and inflammation by inducing the ubiquitination and degradation of IFN-β [223]. Some E3 ligase inhibitors that target the members of the Nedd4 family have been used to treat tumors, and we will discuss the efficacy of these drugs.

ITCH regulates immune response and cancer progression [224,225,226,227]. The results of a high-throughput screening method reported that clomipramine, an ITCH inhibitor, could inhibit the growth of breast cancer, prostate cancer, and bladder cancer cell lines. In addition, clomipramine could kill cancer cells by blocking autophagy [191]. Together, the findings indicate that ITCH inhibitors play a vital role in tumor immunotherapy, providing valuable information for the development of new immunotherapy methods with potential clinical applications. Future studies should examine the crosstalk between ITCH and immunity and design specific ITCH inhibitors to better understand and target ITCH signal molecules in human cancer and improve the effect of tumor immunotherapy.

WWP2 is an E3 ligase associated with carcinogenesis and spread [194], which can cause the ubiquitin-dependent degradation of specific tumor suppressor proteins (such as Oct4 [228] and PTEN [229]) in many cancers [230]. The role of WWP2 in various cancers is not the same. In addition to its carcinogenic effect on the formation of many types of tumors, WWP2 inhibits the proliferation and growth of tumor cells in ovarian cancer [231]. Therefore, WWP2 inhibitors should be cautiously used according to the individualized treatment strategy of patients with cancer. Watt et al. discovered the first generation of WWP2 ubiquitin ligase inhibitors, such as Compound 20 [194], by using a high-throughput screening method based on small-molecule libraries, thus providing a direction for the development of new ubiquitin ligase inhibitors. Although many inhibitors have shown benefits in tumor treatment, more research is required to determine whether they affect tumor immunity.

In addition to the aforementioned specific inhibitors of individual HECT-type E3 ligases, small-molecule inhibitors with broad specificity for these ligases, such as heclin, exist. Thomas and colleagues found heclin while screening the cysteine oxidation catalyzed by the targeted HECT domain. This small-molecule inhibitor can cause conformational changes in the HECT domain and extensively inhibit the activity of HECT-type E3 ligases, thus exerting its antitumor effect [198]. Considering that E3 ligase can affect the activation of the immune system, this compound with broad inhibition may treat tumors by affecting tumor immunity. Therefore, we have listed some HECT-type E3 ligase inhibitors that exhibited satisfactory clinical outcomes; however, whether they affect tumor immunity still needs further exploration (Table 1).

#### 4.1.3. RBR-Type E3 Ligase Inhibitors

To date, RBR is the least frequently targeted class of E3 ligases among inhibitors [3]. The linear ubiquitin chain assembly complex (LUBAC), composed of three subunit proteins (HOIP, HOIL-1L, and SHARPIN), is a polyprotein E3 ligase of the RBR family [232]. LUBAC activity and mutation are associated with diffuse large B-cell lymphoma [3]. The small-molecule inhibitors HOIPIN-8 [9], BAY11-7082, and gliotoxin, as well as stapled helical alpha-peptides targeting the HOIP/HOIL-1L and HOIL-1L/SHARPIN interfaces, can inhibit the activity of LUBAC [232]. Gerlach et al. reported that LUBAC may regulate immune signals [233]. Although no RBR-type E3 ligase inhibitors related to tumor immunotherapy have been identified, a relationship between RBR-type E3 ligases and antitumor immunity may exist. Bendamustine was identified as a selective inhibitor of HOIP based on MALDI-TOFE2/E3 ligase detection [234]. In addition, fragment-based covalent ligand screening could rapidly identify the inhibitors of HOIP active sites [232]. The recently developed strategies involving the use of single-domain antibodies are optimized for HOIP inhibitors and act as crystallization chaperones, thus helping obtain ligand-binding structures to assist in the development of RBR-type E3 ubiquitin ligase inhibitors [235]. Together, these data suggest that targeting RBR-type E3 ligases can be a feasible strategy to screen and develop novel E3 ligase inhibitors for tumor therapy. Future studies should identify new types of RBR-type E3 ligases involved in antitumor immune regulation and clarify their functional mechanisms.

### 4.2. E3 Ligase Agonists

In addition to E3 ligase inhibitors, some E3 ligase agonists (summarized in Table 2) have also been found to promote antitumor immunity.

#### 4.2.1. CRBN

CRBN (cereblon) is a substrate recognition subunit of the CRL4^CRBN^ E3 ligase complex. Thalidomide and its derivatives lenalidomide and pomalidomide are the representative drugs of CRL agonists and mainly target CRBN protein [236,239]. They bind to CRBN and change the substrate specificity of the CRBN E3 ligase complex, leading to the ubiquitination and degradation of downstream proteins (such as Ikaros and Aiolos) and stimulating T-cell activation [236,237]. They are widely used in the clinical treatment of lymphoma and myeloma, such as multiple myeloma [238]. CC-90009 is a newly identified cereblon E3 ligase modulator. CC-90009 combined with CRL4^CRBN^ could selectively target GSPT1 for ubiquitination and proteasome degradation, which can be used to treat acute myeloid leukemia [240,241].

#### 4.2.2. β-TrCP

β-TrCP is the E3 ligase responsible for the polyubiquitination and degradation of nonglycosylated PD-L1 [30,36]. Resveratrol is an E3 ligase modulator that regulates PD-L1 ubiquitination. It induces nonglycosylated PD-L1 polyubiquitination and destabilization by targeting the β-TrCP E3 ligase, thus reducing the expression of PD-L1 in triple-negative breast cancer cells and enhancing antitumor immunity [211,246]. A recent study reported that resveratrol can enhance antitumor T-cell immunity by targeting PD-L1 glycosylation and dimerization [247]. In addition, a study designed various small molecules that could enhance the interaction between SCF β-TrCP and the carcinogenic transcription factor β-catenin, including NRX-252114 and NRX-252262 [242]. These compounds reveal the feasibility of enhancing the E3 ligase–substrate interaction to target the degradation of certain carcinogenic proteins.

### 4.3. Other Drug Development Based on E3 Ligases: PROTACs and Molecular Glue

#### 4.3.1. Proteolysis Targeting Chimeras (PROTACs)

**Figure 4:**
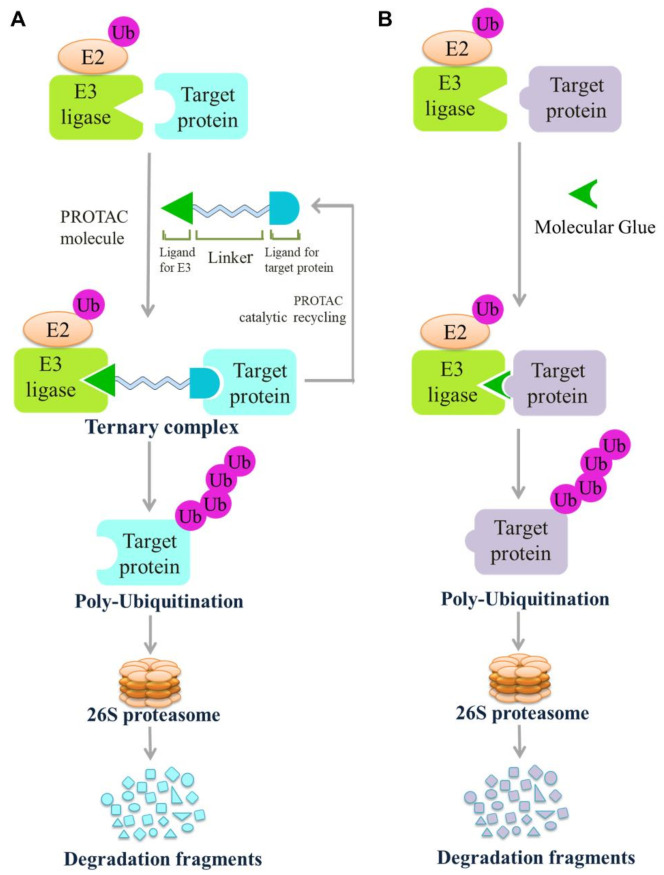
Schematic of PROTACs and molecular glue mechanism. (**A**) PROTAC acts as a medium for inducing E3 ligases to successfully “hold hands” with the target protein, thus forming a stable E3 ligase–PROTAC–target protein ternary complex to induce ubiquitination and proteasome degradation of the target protein. (**B**) Molecular glue, similar to “double-sided glue,” degrades the target protein by inducing or stabilizing the interaction between E3 ligases and the target protein. Some of the graphics in this figure was created using ScienceSlides software 2016 edition (VisiScience Inc., NC, USA).

In recent years, PROTACs, which were first reported by Sakamoto [248], have emerged as a potential therapeutic strategy for cancer [249]. PROTACs can be applied to those undruggable targets to remove unwanted or damaged proteins [249]. This technology links the target protein with E3 ligases through a chemical linker, similar to a dumbbell structure, and forms a stable target protein/PROTAC/E3 ternary complex that induces ubiquitination of the target protein and degradation of the proteasome (Figure 4). Khan et al. reported that the binding of E3 ligases to PROTACs can stabilize tumor suppressor proteins and enhance antitumor activity [250]. PROTACs have become a hotspot in the field of anticancer drug research based on E3 ligases. In addition, PROTACs can potentially enhance antitumor immunity by inducing the presentation of peptides derived from target protein degradation to APCs, thus highlighting the capability of PROTAC compounds in discovering and generating new targets for immunotherapy [251].

The original PROTAC found in *Xenopus laevis* extracts exhibited ternary complex formation (substrate-PROTAC-E3 ligase), ubiquitination activity, and target protein degradation [252]. More than 600 E3 ligases have been found in the human genome; however, only a few E3 ligases have been used in PROTAC designs for various cancer targets, including CRBN, VHL, IAP, MDM2, and β-TrCP [250,253,254,255,256]. At present, PROTACs have been used for target proteins, including hormone receptors (estrogen receptors and androgen receptors), bromine-containing domains, and protein kinases [257]. The first PROTAC drug to enter clinical trials is ARV-110, an oral small-molecule targeting the androgen receptor, which entered a phase 1 clinical trial for the treatment of metastatic castration-resistant prostate cancer in 2019 [256,258] (NCT03888612, Arvinas). In addition, Arvinas developed the PROTAC drug ARV-471 for the treatment of breast cancer. ARV-471 is a degradation agent targeting the estrogen receptor that has entered a phase 2 clinical trial (NCT04072952). The BET protein family, including BRD2, BRD3, and BRD4, play an important role in cancer, among which BRD4 protein is involved in regulating cancer and inflammatory processes [259]. Researchers found that a PROTAC molecule MZ1 can form the BRD4-MZ1-VHL ternary complex, which can mediate the degradation of BRD2, BRD3, and BRD4 proteins [260]. Based on the crystal structure of MZ1, AT1 was developed, which can specifically target BRD4 degradation [261]. In addition, other studies have developed bivalent PROTACs named MT1, which can bind two BRD4 proteins and have a more efficient ability to degrade BRD4 proteins [262]. Recently, a trivalent PROTAC, SIM1, has been designed with higher degradation efficacy and more potent anticancer activity [263].

With the development of PROTACs, some challenges remain to be addressed in clinical applications, including drug resistance, off-target effects, cell permeability, stability, large molecular weight, and difficult synthesis of hybrid molecules [264]. In addition, because of the hook effect, the saturated dose of free PROTAC molecules antagonizes the binding of the binary PROTAC-protein complex and its ternary partner, preventing catalytic degradation [256,265]; the dose issue in the clinical application of PROTACs is also worthy of research attention. Theoretically, PROTACs can induce the degradation of almost all proteins as long as the target protein has a specific ligand available. The main challenge is to identify more specific E3 ligases and ligands for these undruggable proteins to assemble different PROTACs [256]. Because more than 600 E3 ligases are present in the human genome, they can be used to assemble many PROTACs. The use of PROTACs in cancer therapy is promising. To overcome problems in the clinical application of PROTACs, future studies should focus on determining more strategies to develop safer and more effective PROTACs.

#### 4.3.2. Molecular Glue

Molecular glue degraders induce a new interaction between E3 ligases and a target protein, thereby degrading the target protein (Figure 4) [266]. Examples of molecular glues that induce target proteolysis include the immunosuppressant cyclosporine A (CsA), FK506 (tacrolimus), and thalidomide [267]. Compared with traditional small-molecule enzyme inhibitors or receptor antagonists, the most prominent advantage of molecular glue is that it does not require the presence of activity-related pockets on the target protein [268]. Therefore, it can degrade ligand-free proteins, thus targeting more proteins.

Molecular glue can promote the interaction between approximately 600 E3 ligases and more than 20,000 potential target proteins, thus providing a direction for the exploration of new targets and potential small-molecule drugs [268]. Asukamycin, a member of manumycin polyketides, acts as molecular glue between UBR7 and p53, targeting the interaction between the E3 ligase UBR7 and the tumor suppressor p53 in breast cancer cells and resulting in p53 transcriptional activation and cell death [269]. In an earlier study, p53-dependent genotoxic stress–mediated DD1α interacted with T-cells to suppress the immune response and evade immune surveillance [270]. Thus, molecular glue may affect tumor immunity by targeting the interaction between E3 ligases and the substrate protein. Studies have shown that CR8, as a molecular glue degrader, mediates the binding of DDB1-CUL4 E3 ligase to cyclin-dependent kinase 12 (CDK12)-cyclin K, leading to the ubiquitination and degradation of cyclin K [271]. Other studies have found that dCeMM2, dCeMM3, dCeMM4, and HQ461 can also combine with DDB1-CUL4 to degrade cyclin K [272,273]. Thalidomide and its derivatives lenalidomide and pomalidomide have immunomodulatory properties and are used clinically to treat multiple myeloma (MM) [274]. They can recruit zinc finger transcription factors and target proteins to CRBN, leading to their ubiquitination and degradation [275]. Other molecular glues that degrade proteins by binding CRL4-CRBN include CC-885, CC-90009, CC-92480, CC-220, and CC-122 [276,277,278,279,280]. CC-90009 (NCT02848001, NCT04336982) and CC-92480 (NCT03989414) have entered clinical trials. In addition, arylsulfonamide derivatives can also act as molecular glue degraders. For example, indisulam can act as a molecular glue between E3 ubiquitin ligase CUL4-DCAF15 (DDB1 CUL4-related factor 15) and RNA binding protein 39 (RBM39) [243]. Other arylsulfonamide derivatives such as tasisulam, CQS, and E7820 can also degrade RBM39 through a similar mechanism [243,281]. There are also reports that NRX-103094, as a molecular glue, can enhance the degradation of β-catenin peptide by E3 ligase SCF β-TrCP [242]. In addition, some molecular glues that have entered clinical trials include DKY709 (NCT03891953), CFT7455 (NCT04756726), and BTX-1188 (BioTheryX Inc.). DKY709 is in phase I clinical trials as a single agent and in combination with PD-1 antagonists in solid tumors. CFT7455 is used as an oral drug in phase I/II clinical trials of relapsed or refractory non-Hodgkin’s lymphoma or multiple myeloma.

Although molecular glues play a crucial role in drug discovery and development, the identification of most molecular glue degradation products is usually retrospective and accidental [266]. Thus, the discovery of new molecular glues is challenging. With the increased understanding of the mechanism of molecular glue, the discovery of molecular glue has gradually transitioned from accidental discovery to rational design [268]. A recent study identified new molecular glue degraders through scalable chemical profiling [272]. At present, the field of molecular glue is still in its infancy [282]; in particular, its specific effect on tumor immunity remains unclear. More in-depth studies in this field are warranted.

## 5. Conclusions

Tumor immunotherapy currently has the problems of drug resistance and adverse reactions, which limit its clinical application. An effective strategy is to combine other drugs to enhance the efficacy and reduce side effects. Because ubiquitination is involved in multiple processes that regulate tumor immunity, E3 ligases may be a potential therapeutic target for combined tumor immunotherapy. However, in the case of tumor immunotherapy drug resistance, whether E3 ligases can enhance the role of immunotherapy by regulating the role of immune checkpoints and immune pathways remains unknown. In addition, considering the complex and extensive life activities regulated by ubiquitination in the human body, blocking or activating E3 ligases to treat tumors may exert adverse effects on other normal life metabolic activities. Thus, exploring and solving this problem is still a considerable challenge. Moreover, most immune-related pathways can both inhibit and promote tumors. The selection of a suitable therapeutic window to use E3 ligase inhibitors or agonists requires further exploration. Finally, most inhibitors for ubiquitination have been found to be beneficial in preclinical studies but have demonstrated poor results in clinical trials. This difference may be attributable to the insufficient understanding of the structural analysis and medicinal chemistry of the target protein, which requires technological progress.

## Figures and Tables

**Table 1:** The inhibitors of E3 ligases.

Drug Class	Agent	Mechanism	Tumor Types	Phase	Reference
RING-type E3 ligase inhibitor					
IAPs antagonists	LCL161	Smac mimetic, induces degradation of cIAP-1	Advanced solid tumors, hematologic neoplasms	1, 2	[3,156,157]
	APG-1387	Smac mimetic, induces proteasomal degradation of IAPs	Advanced solid tumors, hematologic neoplasms	1, 2	[158]
	Debio 1143 (AT-406)	Smac mimetic, inhibiting XIAP, cIAP-1 and cIAP-2 to promote apoptosis	Advanced solid tumors, lymphomas	1, 2	[159]
	Birinapant (TL32711)	Smac mimetic	Advanced solid tumors, hematologic neoplasms	1, 2	[160]
	AEG40826/ HGS1029	Smac mimetic	Advanced solid tumors	1	[159,160]
	Compound 1 (GDC-0152)	Smac mimetic, binds to the BIR3 domains of cIAP1, cIAP2, and XIAP	Solid tumors	1	[161]
	Compound 13 (AEG40730)	Smac mimetic, binds to the BIR3 domains of cIAP1, cIAP2, and XIAP	N/A	Preclinical	[159]
MDM2 antagonists	AMG 232	Binds to MDM2 and inhibits the MDM2–p53 interaction	Advanced solid tumors, hematologic neoplasms	1	[3,162,163,164,165]
	APG-115	Targeting MDM2-p53 pathway	Advanced solid tumors, hematologic neoplasms	1, 2	[164,166]
	RG7112	Binding to the p53 pocket on MDM2, effectively inhibits the MDM2-p53 interaction	Advanced solid tumors, hematologic neoplasms	1	[3,164]
	SAR405838 (MI-77301)	Binds selectively to HDM2, an oral spirooxindole derivative antagonist of HDM2	Neoplasm malignant	1	[167]
	Idasanutlin (RG7388)	Blocking the MDM2–p53 interaction to reactivate the p53 pathway	Advanced solid tumors, hematologic neoplasms	1, 2	[168]
	Nutlin-3a	Inhibits the MDM2-p53 interaction, leading to p53 stabilization and activation of the p53 pathway	N/A	Preclinical	[169,170]
	HLI98	Inhibits HDM2’s E3 activity	N/A	Preclinical	[171]
	MEL23, MEL24	Inhibits the E3 ligase activity of the Mdm2-MdmX complex.	N/A	Preclinical	[172]
pVHL antagonists	Compound 15, Compound 7, VH298	The targeting of VHL disrupts the interaction of VHL with HIF-α	N/A	Preclinical	[173,174]
SKP2 antagonists	Compound A	Blocks the assembly of Skp2 into the SCF complex.	N/A	Preclinical	[175]
	C1, C2, C16, C20	Inhibits Skp2-Cks1-p27 interface and thereby inhibit p27 ubiquitination.	N/A	Preclinical	[176]
	Compound 25	Prevents the formation of the Skp2-Skp1 complex and inhibits the activity of SCF-Skp2.	N/A	Preclinical	[177]
	DT204	Reduces the binding of Skp2 to Cullin-1 and Commd1, a Cullin-1-binding protein, therefore decreasing SCFSkp2 ubiquitin ligase activity	N/A	Preclinical	[178]
	Betulinic acid (BA)	Binding to Skp2 decreases its stability by disrupting Skp1-Skp2 interactions, thereby inhibiting the Skp2-SCF E3 ligase and promoting the accumulation of its substrates	N/A	Preclinical	[179]
	Dioscin	A new Skp2 inhibitor	N/A	Preclinical	[180,181]
	Curcumin, Quercetin, Lycopene, Silibinin, Epigallocatechin-3-gallate, Vitamin D3	Natural agents that inhibit the expression of Skp2 in human cancers	Variety tumors	1,2,3,4	[7]
β-TrCP antagonists	Erioflorin	Inhibits the interaction of Pdcd4/β-TrCP1	N/A	Preclinical	[182]
	GS143	Inhibits β-TrCP1 ubiquitination of IkB, suppresses NF-kB signaling	N/A	Preclinical	[3]
	UBP-036	Competitive inhibition of substrate binding to β-TRCP	N/A	Preclinical	[183]
Fbxo3 antagonist	BC-1215	Disrupts the interaction of Fbxo3 with Fbxl2	N/A	Preclinical	[184]
Met30 (yeast) antagonist	SMER3	Inhibits SCF-Met30 effectively and selectively	N/A	Preclinical	[3]
Cdc20 antagonists	Tosyl-l-arginine methyl ester	Blocks the APC/C-Cdc20 interaction	N/A	Preclinical	[185,186]
	Pro-TAME	Disrupted the APC-Cdc20/Cdh1 interaction to reduce APC activation	N/A	Preclinical	[187]
	Apcin	Binds to Cdc20 and inhibits APC/C-dependent ubiquitylation	N/A	Preclinical	[185,187]
	Withaferin A	Suppresses Cdc20 activity	N/A	Preclinical	[187]
	NAHA	Inhibits the expression of Cdc20	N/A	Preclinical	[187,188]
	Ganodermanontriol (GDNT)	Inhibits cell proliferation via targeting Cdc20	N/A	Preclinical	[187,189]
TRAF6 antagonist	C25-140	Reduces TRAF6 E3 ligase activity by interfering with the TRAF6–Ubc13 interaction	N/A	Preclinical	[190]
HECT-type E3 ligase inhibitor					
Itch antagonist	Clomipramine	Blocks p73 ubiquitylation by binding to ITCH and inhibiting its charging with Ub	N/A	Preclinical	[191]
NEDD4-1 antagonist	Indole-3-carbinol (I3C) analogues	The potent small molecule inhibitors of NEDD4-1 ubiquitin ligase activity	Adult solid tumor	1	[192,193]
WWP2 antagonist	Compound 20	Binds to the WWP2 HECT domain	N/A	Preclinical	[194]
SMURF1 antagonists	Bortezomib	Downregulated the protein level of SMURF1 by inhibiting SMURF1 mRNA levels	Neoplasm Malignant	1, 2, 3, 4	[195]
	HS-152	Blocked SMURF1-mediated RHOB ubiquitination	N/A	Preclinical	[196]
NEDD4 antagonist	Nitidine chloride	A promising inhibitor of NEDD4	N/A	Preclinical	[197]
Non-specific HECT antagonist	Heclin	Induces conformational change in HECT domain to inhibit activity	N/A	Preclinical	[198]
HUWE1 antagonists	BI8622, BI8626	Inhibits HUWE1 to stabilize assembly of Myc-repressive MIZ1 complex on Myc-activated target genes	N/A	Preclinical	[3]
E6AP antagonists	Compound 12	Inhibits E6AP–p53 interaction	N/A	Preclinical	[3]
	Lutolein, CAF024	Binds to viral E6 protein and prevents its association with E6AP	N/A	Preclinical	[3]
	Lig1, Lig2, Lig3	Inhibits E6-E6AP interaction	N/A	Preclinical	[199]
	N-acetyl phenylalanine	Prevents the trimerization of E6AP and inhibits its E3 functionality	N/A	Preclinical	[3]
	CM11-1	Prevents the poly-ubiquitination of Prx1 and p53 by E6AP	N/A	Preclinical	[3]
RBR-type E3 ligase inhibitor					
LUBAC antagonists	HOIPIN-8	Inhibits LUBAC activity and suppresses linear ubiquitination-mediated NF-κb activation.	Human lung carcinoma A549 cells and HEK293T cells	Preclinical	[9]
	BAY11-7082	Inactivates the E2-conjugating enzymes Ubc13 and UbcH7 and the E3 ligase LUBAC	pre-B ALL, natural killer/T-cell lymphomas, gastric cancer	Preclinical	[18,200]
	Gliotoxin	Inhibits LUBAC and suppresses NF-κB activation	N/A	Preclinical	[201]
	Stapled peptides	Inhibits LUBAC through the disruption of the HOIL-1L-HOIP interaction and loss of the functional complex	N/A	Preclinical	[202]
HOIP antagonist	Bendamustine	Specifically inhibits HOIP	Solid tumors, hematologic neoplasms	FDA approved (Phase 4)	[3]
N/A: not applicable					

**Table 2:** The agonists of E3 ligases.

Drug Class	Agent	Mechanism	Tumor Types	Phase	Reference
E3 ligase agonists					
Cereblon (CRBN) agonists	Lenalidomide, Thalidomide, Pomalidomide	Modulation of the substrate specificity of the CRL4-CRBN E3 ubiquitin ligase, induces the ubiquitination of IKZF1 and IKZF3	Multiple myeloma, diffuse large B-cell lymphoma	FDA approved (Phase 4)	[236,237,238,239]
	CC-90009	Promotes binding of cereblon to GSPT1, leading to enhanced ubiquitination and subsequent degradation	AML, leukemia, myelodysplastic syndromes	1, 2	[240,241]
	CC-122 (Avadomide), CC-220 (Iberdomide)	Cereblon E3 ligase modulators (CELMoDs)	AML, multiple myeloma, diffuse large B-cell lymphoma (DLBCL), advanced solid tumors, non-Hodgkin’s lymphoma (NHL), melanoma	1, 2	[239]
β-TrCP agonists	NRX-252114, NRX-252262, NRX-1532, NRX-1933, NRX-2663, NRX-103094, RX-103095	Promotes the interaction of β-TrCP with β-catenin	N/A	Preclinical	[242]
DCAF15 agonists	Indisulam(E7070), Tasisulam, CQS	Promotes the binding of Rbm39 to DCAF15	Metastatic breast cancer, gastric cancer, leukemia, melanoma (skin), solid tumor, kidney neoplasms, adenocarcinoma, CRC	1, 2	[243]
TIR1 agonists	Hormone auxin	Binds to SCF F-box subunit TIR1 and promotes the interaction between TIR1 and its substrate	N/A	Preclinical	[244]
NPR agonists	Aalicylic acid (SA)	Regulates the effect of CRL3-NPR	N/A	Preclinical	[244,245]
COI1 agonists	Jasmonic acid (JA)	Facilitates the molecular association between SCF-COI1 ligase and its substrates	N/A	Preclinical	[244]
N/A: not applicable					

## Data Availability

Not applicable.
